# Health Promotion in Israeli Colleges of Higher Education—The Example of Oranim College of Education

**DOI:** 10.3389/fpubh.2020.00408

**Published:** 2020-09-17

**Authors:** Irit Hof-Nahor, Seema Biswas

**Affiliations:** ^1^Oranim Academic College, Kiryat Tiv'on, Israel; ^2^Department of General Surgery, Galilee Medical Center, Nahariya, Israel

**Keywords:** teacher-training, health promotion, schools, education college, exercise

## Abstract

A teacher-training college that promotes a healthy learning environment contributes to the educational character of teachers of the future. These teachers adopt healthy lifestyles of their own and influence the lifestyles and learning of their pupils in kindergartens and schools. This article outlines the implementation of a health promotion program in a teacher-training college in northern Israel—Oranim College of Education. We describe the changes made at the college over three years—from the commitment of college executives to the health promotion policy initiative launched by the Ministry of Education, the appointment of a health leader (health promotion coordinator), staff and student engagement in change, rededication of college resources to healthy lifestyles and learning, promotion of healthy eating and exercise, changes in teaching, new courses and more interaction with the community, to health promotion by teachers in training in local kindergartens and schools. Using real examples, we show how effective policy implementation rests on the active participation of all stakeholders.

## Introduction

Health is defined as the balance of physical, emotional, social, spiritual, and intellectual health, rather than simply the lack of disease (1). Health promotion is an active process that allows people to increase the control they exert over their own health. In response to a worldwide increase in awareness of the importance of public health, the Ottawa Convention in 1986 and subsequent Charter (2) marked a turning point in health promotion in all sectors of public life and work. The Charter emphasized five active strategies: framing healthy public policies; creating supportive environments; promoting community activities; strengthening individual skills; and reorganizing health services (3). Universities and colleges have embraced the first four of these strategies, seeking to improve the health and life-style choices of staff and students. The Okanagan Charter (4) and the National Association of Student Personnel Administrators in higher education (NASPA) and the National Intramural and Recreational Sports Association (NIRSA) statement on well-being in higher education (5) affirm the commitment of higher education institutes in health promotion to their staff, students and society at large. In this article we describe the implementation of the Israel Ministry of Education's health promotion policy in one of the foremost teacher-training centers of higher education in the country—Oranim College.

Oranim College of Education is the largest and leading academic college of education in northern Israel. College students and staff represent the mosaic of Israeli society, with secular and Haredi Jews, Arabs, Bedouin, Druse, Ethiopians and migrants from across the world. The college may, consequently, be regarded as a microcosm that reflects the diversity of Israel's population. Graduating with Bachelor and Master of Arts, Sciences and Education degrees, the potential influence of an effective health promotion strategy on qualifying teachers working in kindergartens, elementary and high schools is far reaching. Commitment of college staff to the concept of active health promotion has been central to the sustainability of the program, and Oranim's roots in the kibbutz movement, proximity to Haifa (Israel's third city and high-technology hub) and the small agricultural (and, in some cases, increasing socially disadvantaged) communities of the Zevulun district, form the foundational basis of health promotion strategies embedded in local multi-ethnic culture.

The number of students currently studying in the first year of their first degree is 2,320 and second degree is 540. Over 83% of students are women. Twenty percent of women studying their first degree and 64% in their second degree are married. Similarly, 20% of men in their first degree and 90% of men in their second degree are married. The average age of undergraduate students is 24 years and of graduate students is 38 years. Most undergraduate students (95%) are Israel-born, at least 30% of whom have registered as coming from minority ethnic backgrounds.

There are 600 academic staff. Their average age is 55 years and ~70% are women. There are 200 administrative staff. Their average age is 36 years and ~85% are women.

As a center that educates educators, the approach of the college has been to provide individuals (students and staff) with the knowledge and skills essential to decision-making that favors healthy lifestyle choices through increased health awareness rather than incentive or inducement (2). This has required the provision of health promotion environments and activities that are practical, achievable, and well-received. A college health lead (a voluntary appointment) was tasked with the selection and coordination of a steering team representing the entire college community: senior management; academic and administrative staff; and, student representatives (the chair of the student union). Tsouros et al. (6) describe a similar organizational structure in the forum assembled for health promotion in higher education in the UK. The Oranim College health lead, the main author of this paper, is an academic member of the Faculty of Natural and Environmental Sciences, a teacher, researcher and experienced manager of multiple programs at the college for over a decade—thus, familiar with key positions at the college, the mission and culture of the college, and student and staff demographics.

## Policy Options

In 2016, a forum was assembled at the Mofet Institute, a consortium of colleges of education for research and development of policy in teacher education, in order to implement a national health promotion program for Israeli schools and kindergartens. A task force composed of officials of the Ministry of Education and college academics was put together to establish a structured process for health promotion in Israeli academic institutions, with guiding standards for implementation.

The forum set about building consensus between multidisciplinary professionals in health education and teacher education. The consensus was created using a framework of qualitative research tools: in-depth discussions, dialogues and presentations of experts' positions, with continuous feedback from the academic field. With the infrastructure of each education college in mind, standards were set for health promotion goals specific to each college with criteria to measure success in implementation. A proposal was published on the Ministry of Education website as an online application for health promotion. All education colleges in the country were eligible to apply. Colleges judged to have successfully implemented effective health promotion programs were to be accredited as education establishments with active health promotion programs. A committee comprising forum members was set up to evaluate performance in policy implementation and award accreditation (granting between 1 and 3 stars). Evaluation of each college is annual, and findings are available to all members of the consortium to use as guidance for implementation and improvement of their own programs.

Criteria for the first star awarded relate to the commitment of the management of each college to integrate health promotion into the overall vision of the college—creating a physical environment that promotes health on campus. A second star relates to teaching and ongoing campus health promotion activities. A maximum of two stars were to be awarded after initial evaluation (2 years after implementation of the program). Three stars are awarded after health promotion program evaluations within the institutions themselves are complete, and health promotion material and activities have been shared with other campuses, as well as local schools and kindergartens and the wider community.

Five of almost thirty colleges of education in Israel accepted the initial proposal, appointed a health leader (health promotion coordinator) and began development of college health promotion programs. We describe the implementation of health promotion activities in Oranim College over the last 3 years.

## Policy Implications and Implementation

### Vision and Creation of an Active Health Promotion Environment on Campus

The senior management of the college (the president and rector) signed a declaration of serious intent to integrate health priorities into the college's vision for teacher-training. This mission statement has become integral to how the college functions and is marketed. The health promotion objectives embedded within the delivery and content of the curriculum endow the college with a competitive advantage in student applications for training—students prefer to study in a healthy environment.

Health promotion posters are hung in corridors and central transit areas. College emails with weekly health promotion messages and details of health promotion lectures open to everyone are sent and become the topic of the day—frequently incorporated into daily lessons. Efforts to estimate the impact of these posters, emails and open lectures, showed that the number of student email responses to the College Health Coordinator in 2019 had doubled in comparison to the same months in 2017 (Table 1).

**Table 1:** Email interest in health and nutrition (December, March, and June are the 3 months where there is daily student attendance—no college holidays).

	**December**	**March**	**June**	**Total**
2017	20	27	18	65
2019	42	38	56	136

### College Nutrition

The college provides clean areas for heating and eating food that students bring from home (staff and students, thus, eat healthier and cheaper meals of their own making).

More microwaves, water coolers and water heaters have been purchased. The use of these areas and equipment is free of charge. The areas are cleaned several times a day, and equipment is maintained and serviced by the college.

There are three rooms equipped and appropriately furnished for nursing mothers to express and refrigerate breast milk in a pleasant environment (over 80% of students are women).

In their report on the effectiveness of subsidizing healthy meals at the Harvard School of Public Health, Michels et al. (7) describe a significant change in individual food choices toward a healthy diet as a result of health education and cost reductions of healthy food items in the cafeteria. More importantly, this change persisted and grew further even after these price reductions were discontinued. Our decision in Oranim College was to replace the entire menu of the cafeteria. The new menu is more varied. The proportion of fried or processed food has been significantly reduced. Food is now prepared onsite; there are more salads and cooked meals. Fresh fruit, vegetables, legumes and bread are purchased locally. The meals are subsidized by the college, and prices have not increased. The student union has initiated a “Give & Take Market” where organic fruit and vegetables grown by students and staff are sold.

The composition of refreshments served at college events and ceremonies has changed drastically. Before implementation of the health promotion program in 2016, over 85% of the refreshments comprised a variety of sweet and savory pastries and sandwiches served on disposable plates with plastic utensils. Only 15% of the food served included fresh fruit. No fresh vegetables or salads were served. With the appointment of a new college caterer in 2017, over 60% of the refreshments now include a variety of fresh salad and fruit. Twenty percent are fresh quiches or stews, 10% are yogurts and 10% sweet desserts, such as freshly baked cakes. In an initiative to involve socially disadvantaged sections of the local community, in 2019, a “Local Women's Cooking” group (http://cooking-woman.co.il/women-cooking/) undertook the catering at a number of college gatherings. The Cooking Group has its origins in the diversity of the Jewish diaspora returning to Israel with their own “ethnic culinary roots.” In Oranim College this group also comprises local Arab women of diverse backgrounds and culinary roots.

The current catering budget has seen an increase of over 30% compared with that of 2016. Table 2 gives some examples of food items resulting in the budget increase. To place these prices in context, similar salads (between 200 and 250 g portions) in a popular national restaurant chain cost between 40 and 65 NIS (12 and 19 USD).

**Table 2:** Examples of prices of common items in the college catering budget.

**Food item**	**Price**
**2016**	
12 units of white bread sandwiches 1 kilo (40 units) of pastries	85 NIS (25 USD) 30 NIS (9 USD)
**2019**	
10 units of whole wheat bread sandwiches Vegetable salad (about 4 kg) Green leaf salad (about 4 kg)	100 NIS (30 USD) 120 NIS (35 USD) 120 NIS (35 USD)

### Outdoor Activity

The college is located within a green landscape with a celebrated botanical garden, walking paths and areas to congregate, teach and learn. While previously overlooked by students and busy staff, the walking paths have now been inaugurated for the benefit of the entire college community as greenery has been shown to be effective in improving cognition among students, promoting an effective learning environment and reducing stress (8, 9). The botanical garden spans 10 acres and has 900 different plant species (most of them non-cultivated and indigenous to Israel). Each section of the garden represents a unique habitat or geographical region. Two walking paths: the Poetry Path where verses from famous poems are displayed on signs along the route; and, the Biomimetic Path with nine educational stations on natural history form an ideal environment for exercise and for senior students to teach children from local underprivileged communities.

College events and ceremonies are now held in these open spaces. Over the last 3 years, biology, immunology, ecology and art classes have been conducted in this “open” classroom. Courses on growing healthy food and recognizing healthy wild flora and fauna are held for staff, students and local children. Students learn to cultivate their own vegetables in the college gardens, and, on weekends, family workshops are offered for students, staff and local families. Table 3 shows the increase in outdoor college activity since implementation of the health promotion program.

**Table 3:** Outdoor college activity—the proportion of events held outdoors is given as a fraction of the total held outdoors and indoors on campus.

	**2016**	**2019**
Events organized by the Student Union	4/8	8/11
Official college Ceremonies	8/10	10/12
Study days	12/18	18/23
Classes	17/68 spring semester 7/67 winter semester	51/68 spring semester 27/67 winter semester

Students are increasingly walking between classes. College security staff report a 40% reduction in shuttle use around the campus over the last 3 years—a cost saving of 50 000 NIS (15 000 USD) per year. Morning shuttle use, however, has remained the same with a peak between 8 and 8.30 a.m.—the first class begins at 8.15 a.m.

### Curriculum

In keeping with health promotion changes in higher education academies worldwide, teacher training now incorporates seminars on the importance of nutrition on brain development and learning in childhood (10–12), and compulsory and selective courses on the importance of learned health behaviors and informed health decisions (13, 14) have been developed. These lecture courses cover diverse health issues including: family health; nutrition; eating habits; the effects of smoking; importance of physical exercise; hygiene; vaccination; the immune system; mental health; and, women's health. In response to student feedback, health-related topics have been incorporated into existing courses and new health-related courses such as Family Health Promotion, My Personal Health and Sustainability in Health developed. Whereas, in the past health topics only comprised 20% of a typical lesson; in some courses health topics now comprise up to 80% of a lesson. The courses are tailored for pre-service as well as working teachers (Table 4).

**Table 4:** Courses related to health promotion (including new courses introduced in 2016).

**Course**	**Faculty**	**Compulsory/ Selective**	**Credit points**	**Duration**	**Students eligible**	**Number of registered students**	
						**2016/2017**	**2019/2020**
Food for thought	Faculty of Natural and Environmental Sciences	Selective	6	14 weeks	Pre-service teachers	22	19
Sustainability and Health	Faculty of Natural and Environmental Sciences	Selective	6	14 weeks	Pre-service teachers	–	12
Health promotion	Faculty of Advanced Studies	Compulsory	2	14 weeks	Elementary Mathematics and Science for Teachers	24	28
Brain development in early childhood	Faculty of Natural and Environmental Sciences	Selective	6	14 weeks	Pre-service teachers	21	26
My personal health	All faculties	Selective	2	14 weeks	All	68	107
My family's health	Faculty of Education	Selective	2	14 weeks	Kindergarten teachers	–	9
Family health promotion	Faculty of Education	Selective	2	14 weeks	Pre-service Kindergarten teachers	14	21
Women's health in the modern world	Faculty of Natural and Environmental Sciences	Selective	2	14 weeks	Pre-service teachers	18	–
Introduction to nutrition	Faculty of Natural and Environmental Sciences	Selective	2	14 weeks	Science students only	10	25
Mindfulness in education	Social Sciences and humanities	Selective	2	28 weeks	All	–	20
Health education	Faculty of Natural and Environmental Sciences	Compulsory	6	14 weeks	Pre-school science teachers	–	29
Total students learning						177	306

Pre-service teachers now incorporate health promotion (dental hygiene and cigarette smoking, for example) directly or and indirectly into their lessons in local kindergartens and schools—teaching 12 lessons throughout their final year. Since 2019, at least 8 of these lessons cover health education issues (in contrast to 4–6 lessons in previous years). These training lessons are evaluated by college academics, and, in keeping with similar health promotion initiatives in schools worldwide (15, 16), feedback from teachers in training is that their confidence in incorporating health promotion into school lessons has grown (Table 5). Topics vary from understanding food chains to the importance of exercise, sleep and healthy eating for children and their families. In order to embed these lessons within local school curricula, they are increasing linked to national or international calendar landmarks such as Family Day, Sports Week, World Health Day and World Diabetes Day.

**Table 5:** Feedback from teachers in training on Oranim health promotion courses.

**Course Name**	**Number of students on course**	**Number of students who completed course feedback**	**The course contributed to my knowledge of health issues**	**The course affected my views on healthy life choices**	**The course has changed my view of my family's health**	**On this course I learned strategies to promote health in my teaching**	**As a result of this course I am more confident that I can teach health issues**
Introduction to nutrition 2016	10	10	9 (90%)	8 (80%)	5 (50%)	5 (50%)	8 (80%)
Introduction to nutrition 2019	25	13	11 (85%)	13 (100%)	8 (73%)	9 (81%)	11 (85%)
Health education 2018	29	21	18 (86%)	17 (80%)	17 (80%)	12 (57%)	9 (43%)
Food for thought 2016	22	14	9 (64%)	12 (86%)	5 (36%)	8 (57%)	10 (71%)
Food for thought 2019	19	18	17 (94%)	15 (83%)	7 (39%)	13 (72%)	14 (78%)
Health promotion 2016	24	16	16 (100%)	14 (87%)	10 (62%)	15 (93%)	8 (50%)
Health promotion 2019	28	23	15 (65%)	13 (56%)	7 (30%)	13 (56%)	14 (61%)

The college invites expert speakers on health promotion. Lectures are free and available to the public. Topics include: the health effects of the human microbiome, the importance of physical activity on health throughout our lives, the impact of modern lifestyles on brain development, food waste and food security. Due to the popularity of the lectures, the lecture program has been expanded.

### Physical Activity

The importance of physical activity in terms of healthy lifestyle (17) and in enhancing academic achievement (18, 19) has been extensively researched. Teleman et al. (20), in Italy, found that physical activity was the most important factor in health promotion and well-being among university students. Physical activity in Oranim College is promoted on campus in multiple ways. Walking routes between the study buildings and the botanical gardens have been marked out with walking distances. In the morning there is a 20-min walk through the botanical gardens for staff and students to join. Most lecturers have incorporated periods of physical activity into their lessons. There are Zumba, Pilates, Yoga, and gymnastics classes during the college day and in the evening. These classes are heavily subsidized and supported by the student union. Professional instructors have been engaged by the college and students register their interest for each class. The college has also invested in the renovation of college buildings that house these classes to make them more suitable for sporting activity.

### Engagement With the Community

Twice a year Oranim College invites the community to participate in a program of up to 30 workshops in the fields of science, art and communication. These two community events—Science Day (in March) and European Researchers' Evening (in September)—are attended by up to a 1,000 people from local communities and schools.

Five new activities and lectures dedicated to health issues are now included in Science Day (there were none in 2016). In 2019, 80 people attended the two lectures, and 300 people attended the workshops, two of which took place at the botanical garden. Attendees were mainly families with children and senior citizens of the local community. Feedback from the local community has been strongly supportive with particular mention of the relevance to children and the closeness of the college to the community.

The college has since opened three courses/academic programs (Table 6) also available to the community.

**Table 6:** New community courses related to health promotion (course fees are a guide before deductions are included).

**Name of course/program**	**Faculty**	**Duration**	**Fee**	**Number of participants enrolled**	
				**2017**	**2019**
Forest education	Faculty of Education & Faculty of Natural and Environmental Sciences	4 years	10,000–13,000 NIS per year	–	17
Horticultural therapy	Faculty of Natural and Environmental Sciences	2 years	12,000 NIS per year	12	28
Health education (to open in 2020)	Continuing Education Program for teachers in elementary and high schools	2 months	600/800 NIS	–	–
Total enrolled up to end of 2019				12	45

## Actionable Recommendations

One of the most effective ways to promote health is to integrate health strategies into the fabric of public infrastructure: transport networks; the workplace; recreational spaces; and, we believe, most crucially, into the fabric of educational institutions—from kindergartens, elementary schools, high schools, to colleges, and universities. Educational institutions shape the personalities and decisions of individuals as they grow. Teachers in training and service exert substantial influence on the development and life-choices of their students, as well as being impacted themselves, by a healthier workplace (6, 21–25).

We have described only a few of the changes at Oranim College that have transformed the teaching and learning environment into one that promotes health, not simply for college students and staff, but for pupils taught by teachers in training and new graduate teachers of the college. Not all our initiatives are described. We had some failures: our efforts to reduce the use of personal vehicles floundered as the public transport infrastructure in the region simply cannot support car-free transportation. The college invested over 110 000 NIS (over 30 000 USD) in providing a free scheduled shuttle (and taxi) service for staff and students to the local train station (5.6 km away). The service was underutilized from the outset as students and staff (especially with young families or in part-time employment) were unable to predict their daily schedules, choosing to drive themselves, share rides, or use popular online applications or the student union Facebook page to organize transport. People who had registered for the service failed to materialize, even when private taxis had been ordered for them. A scarcity of trains and delays caused students to miss the shuttle and forced students to opt for private transport.

Complaints of passive-smoking and cigarette litter on college lawns, outside the cafeteria, in the corridors of student dormitories and at building entrances prompted the college to designate specific smoking areas, especially for boarding students (50% of all cigarette and hookah smokers on campus), and institute a no-smoking policy in public areas within the college grounds (as per the national policy for public and workplaces since 1983). Failure to enforce the no-smoking policy, monitor the areas or levy fines, and complaints that the designated areas were too restrictive (especially in the winter) resulted in repeated violations, although, the chief of housekeeping did report a 40% (5 kg) reduction in cigarette butts collected around the campus between 2016 and 2019. The college is examining other alternatives: fitting smoke detectors; implementing disciplinary measures if repeated warnings are ignored; or, even installing cameras in public places. Initial reactions to suggestions of the placement of cameras have served as an important reminder of our philosophy of promoting health through choice rather than inducement or coercion, as these have been met with vigorous opposition. More favorable have been proposals for smoking booths (common in airports) and an anti-smoking health promotion campaign supported by the student union. New intakes of students are to be designated rooms so that smokers share the same dormitory building.

We were unable to completely phase-out fried and processed food from the cafeteria as these remain popular choices on an otherwise fairly restricted menu, and the provision of healthier alternatives has been expensive. Our research of similar initiatives in other university cafeterias has shown that cost-effective healthy menus can work when there is a large volume of diners (including administrative staff, porters, visitors and even bus or taxi drivers whose work brings them to the campus).

Our hope that recycled coffee scraps as fertilizer for the botanical gardens would stimulate students and staff to collect these on their own initiative and take these to the cultivation areas in the gardens were too ambitious. As a result, instead of relying on voluntary separation of organic from general waste in the cafeteria, all catering and housekeeping staff have been recruited to the separation and collection of organic waste. In 2019, 250–300 l of organic waste (weighing ~90–100 kg) was collected—a 30% increase on 2018. This is used for composting in the college gardens. The frequency of coffee waste collection from the cafeteria also increased. We also hoped that the new equipment for students to prepare and heat their own food would be cleaned and maintained by the students. This, too, was too ambitious. The college oversees these processes as they remain an important part of our health promotion program.

Exercise classes (Zumba, Pilates, and Yoga) are canceled if fewer than 6 staff or students arrive for a class. We have found by trial and error and with student feedback which classes are the most popular and when these classes are best held. The student union have been instrumental in promoting the classes and registering sufficient numbers for the classes to continue and maintain the enthusiasm of hired instructors. The results of their efforts are seen in Table 7. The Zumba class was fully booked (20 participants) in 2019, thus, the decision has been taken to run this class twice (at 15:30 and 16:30) on the same day. The cost of every class has remained the same over the last 3 years (15 NIS−4 USD).

**Table 7:** Number of participants in exercise classes.

	**2016**	**2019**
Zumba	8	38
Pilates	–	7
Yoga	10	10
Aerobic	–	13

Similarly, in spite of the success of outdoor courses and ceremonial activities, the number of participants on scheduled morning walks fell from 12 soon after the initiative began in the first semester of 2016, to three by the end of the semester. As a result, the initiative was discontinued.

Our most successful initiatives show what may be achieved through the engagement of college staff and the students themselves, and the importance of commitment to program goals from college executives from the outset. Oranim College was awarded 2 stars in 2018 and, with four other colleges in Israel, received three stars in 2019 after sharing our experience in health promotion with local schools. The first teachers of the college as a health promotion institute have now graduated and are employed in these schools. Health promotion is integral to their teaching and local schools have eagerly embraced this.

We strongly recommend a discussion from the outset at the highest level of college management of the sincerity of commitment to policy goals. These senior colleagues form a steering team. This substantially increases the likelihood of change as support to drive through change becomes implicit amongst administrative staff and staff in control of key budgets. Support “from the top” becomes understood by all college personnel, the students and even the wider community. Colleges that have not appointed steering teams have failed to win star ratings.

The health promotion program has been funded through existing budgets and added investment. We have described specific examples of investment, in the cafeteria and catering facilities, for example, and cited savings in shuttle transport around the campus as students and staff walk more. The true financial impact of the health promotion program on the college is difficult to define as changes have been gradual, initiatives have evolved and new courses and systems have been engineered to incorporate health promotion from the outset. Also hard to evaluate is the impact of social capital comprising not simply the college, but the surrounding community and generations of new teachers.

It was no accident that the coordinator appointed was an experienced, well-respected and popular member of the academic staff. Receptiveness and objectivity are important qualities necessary to assess which initiatives are working and which are failing and why. The coordinator coordinates policy implementation but also coordinates communication and reassures staff and students how changes will affect them, helping them to adapt. Our failure to restrict smoking around the campus and promote public transportation illustrates the importance of student engagement in overcoming barriers to change; while success in the promotion of physical activity and healthy eating were crucially dependent on student (and student union) support.

There remains more to do. Through curricular and extra-curricular college activities the knowledge, skills and attitudes needed to promote a healthy learning and working environment have been embraced. These must be developed further. The health and education benefit passed on to the children taught by generations of Oranim College graduates need to be studied. The children will, in time, add to and improve our efforts, and shape the health of their families and communities.

In 2020, all nine education colleges participating in the health promotion policy forum will evaluate the effectiveness of their individual programmes as staff and students complete anonymous questionnaires. Results from across the country will reveal the wider effects of the policy and provide insights into the effectiveness of local health promotion initiatives.

## Conclusion

Institutions of higher education have the capacity to influence the campus and the wider community they serve. A teacher training college that promotes health invites its teachers in training and future teachers to promote the health of their pupils and the communities they serve.

## Author Contributions

IH-N directed the health promotion program, devised the concept of the paper, wrote the first draft of the paper, and was involved in subsequent revisions. SB contributed to the concept of the paper and rewrote all revisions. Both authors contributed to the article and approved the submitted version.

## Conflict of Interest

The authors declare that the research was conducted in the absence of any commercial or financial relationships that could be construed as a potential conflict of interest.
